# The Progress of PVDF as a Functional Material for Triboelectric Nanogenerators and Self-Powered Sensors

**DOI:** 10.3390/mi9100532

**Published:** 2018-10-20

**Authors:** Jin Pyo Lee, Jae Won Lee, Jeong Min Baik

**Affiliations:** School of Materials Science and Engineering, KIST-UNIST-Ulsan Center for Convergent Materials, Ulsan National Institute of Science and Technology (UNIST), Ulsan 44919, Korea; leejp@unist.ac.kr (J.P.L.); leejw@unist.ac.kr (J.W.L.)

**Keywords:** PVDF, sensor, triboelectric nanogenerator

## Abstract

Ever since a new energy harvesting technology, known as a triboelectric nanogenerator (TENG), was reported in 2012, the rapid development of device fabrication techniques and mechanical system designs have considerably made the instantaneous output power increase up to several tens of mW/cm^2^. With this innovative technology, a lot of researchers experimentally demonstrated that various portable/wearable devices could be operated without any external power. This article provides a comprehensive review of polyvinylidene fluoride (PVDF)-based polymers as effective dielectrics in TENGs for further increase of the output power to speed up commercialization of the TENGs, as well as the fundamental issues regarding the materials. In the end, we will also review PVDF-based sensors based on the triboelectric and piezoelectric effects of the PVDF polymers.

## 1. Introduction

### 1.1. Background of Energy Harvesting Based on the Triboelectric Effect

**Figure 1:**
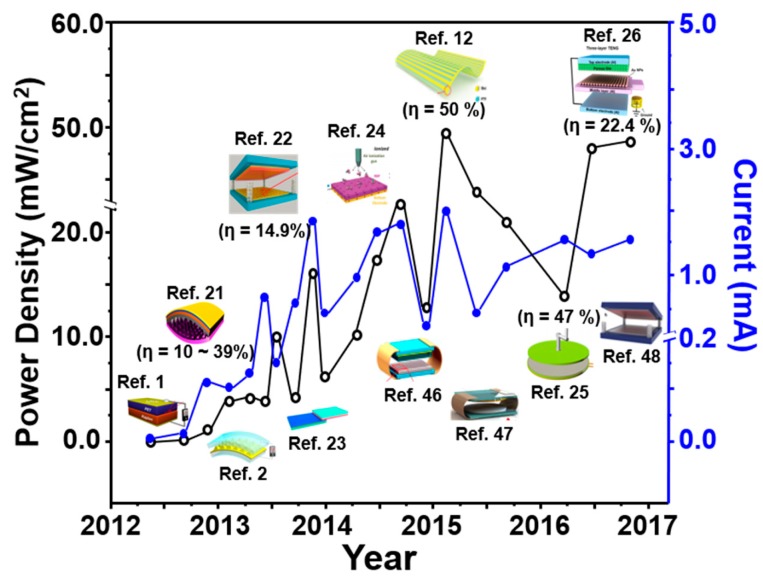
The areal power densities and output currents of various triboelectric nanogenerators (TENGs) reported for the last 5 years [45].

Most recently, a newly designed energy generating device named the triboelectric nanogenerator (TENG) was reported and various types of TENGs have been demonstrated, proven as a highly efficient, simple, robust and cost-effective technique for efficiently converting various mechanical energies around us to electricity [1,2,3,4,5,6,7,8,9,10,11,12,13,14,15,16,17,18]. In principle, the electrical energy is generated as two different materials are brought into contact with each other, in conjunction with the triboelectric electrification and electrostatic induction. The sources of mechanical energy such as winds and moving things are available anywhere and anytime in our surroundings [18,19], thus, TENG will be appropriate as a power-supply unit for portable devices although the energy is not big as expected [20]. Since the first demonstration of the TENG on 2012, various TENG structures and new functional materials brought about the significant increase of the instantaneous power density up to several tens of mW/cm^2^, as shown in Figure 1 and Table 1 [1,2,12,21,22,23,24,25,26,27,28,29]. The output current also increased up to several mA although it seemed that there was no significant increase recently. By virtue of the increased output power, it was successfully demonstrated that it was possible to power up various portable electronic devices as well as to fabricate the self-powered physical and chemical sensors. Actually, many portable/wearable devices such as smart phones/watches, healthcare sensors, smart goggles, etc., could be charged by the TENGs, which generated enough power to turn on or use the devices for a moment. The TENGs also made various sensors which could mechanically detect the ambient pressure [16,30,31,32,33], motion [34], airflow [35], vibration [36], and chemically detect various chemical/molecular species including methanol [37], ethanol [38,39,40], mercury [41], glucose [42], phenol [43], metal ions [44] without any external power.

### 1.2. The Four Fundamental Modes of the Triboelectric Nanogenerator

**Figure 2:**
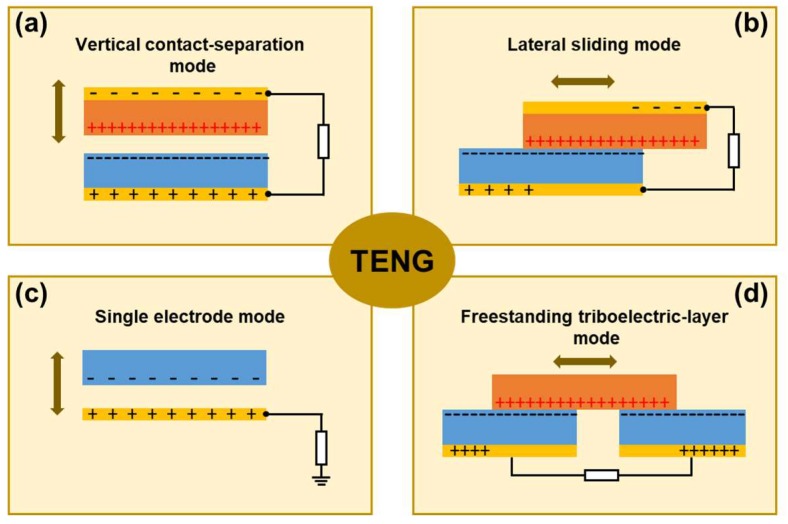
The four fundamental operation modes of the triboelectric nanogenerators. (**a**) The vertical contact-separation mode. (**b**) The lateral sliding mode. (**c**) The single-electrode mode. (**d**) The free-standing mode.

Up to now, there are two representative modes in TENGs such as the vertical contact-separation mode and lateral sliding mode depending on the direction. An object moves with respect to the other one. In the vertical contact-separation mode, the working mechanism of the TENG is understood as the potential difference occurring due to the contact and separation of two surfaces, as shown in Figure 2a. When an external force is applied onto the top surface of the device, the surface comes into contact with the other surface and the charge transfer occurs between them, generating positive charges on the surface of one material and negative charges on the other one. The charge transfer is mainly determined by the intrinsic properties of the materials, known as the triboelectric series. As the force is released, the two surfaces with opposite charges are separated, inducing a potential difference across the top and bottom electrodes. This is the driving force of the electron flow through the external circuit from one electrode to the other one.

In the second operation mode, the two surfaces are brought into contact in parallel with each other, named as the lateral sliding mode, as shown in Figure 2b. Once the top surface with positive charges slides outward, the decrease of the contact area induces the flow of electrons through the external circuit because of the in-plane charge separation generating an electric field parallel to the plates. Subsequently, when the top surface reverts to the original position, the electrons on the top electrode will flow back through the external circuit to the bottom electrode in the same manner. Compared with the vertical contact-separation mode, the lateral sliding mode shows a quite high energy conversion efficiency and is quite promising in wearable application because of the low input energy and various sliding motions occurring during human movement.

Additionally, there were two more operation modes, the single electrode mode, and free-standing mode. Specifically, the single electrode mode has only one electrode, as shown in Figure 2c. In the mode, the surfaces of the polymer and metal electrode are brought into contact with each other and the charge transfer occurs between them. Once the polymer and the electrode are separated, the negative charges on the surface of the polymer induced positive charges on the electrode, driving free electrons to flow from the electrode to the ground, creating a current. The free-standing mode triboelectric nanogenerator can scavenge electrical energy without an attached electrode, as shown in Figure 2d. The TENG consists of two electrodes underneath a dielectric layer and the other frictional layer. The frictional layer approaching to and departing from the electrodes creates an unbalanced charge distribution, causing the electrons to flow between the two electrodes to balance the local potential distribution. It is well-known that this mode could generate quite a high energy by the optimization of the electrode pattern and the proper choice of the materials.

### 1.3. The Importance of Dielectrics in the Triboelectric Nanogenerator

As mentioned above, the instantaneous areal power density has reached up to several tens of mW/cm^2^. However, this does not mean the improvement of the energy conversion efficiency. It tells us facts that the fabricated TENGs can generate the output powers at confined input conditions (i.e., large input force or optimized input conditions). The areal output currents reported are also still less than 1 mA/cm^2^, too low to provide enough energy for electronic devices. Recently, it seems that there was no more significant increase in the output power of the TENGs.

It is well-known that the output current of the TENGs is determined by the density of the surface charges transferred between the two materials. As a positively charged material, aluminum (Al) was widely used because of the excellent electron donor characteristics although there have been promising candidates consisting of Au, Cu, etc. On the other hand, various polymers such as polydimethylsiloxane (PDMS) [49,50], polytetrafluoroethylene (PTFE) [51,52,53], fluorinated ethylene propylene (FEP) [54,55], and polyimide (PI) [56,57,58] have been tested as negatively charged materials. Among many polymers, polyvinylidene difluoride (PVDF) has many advantages such as a large dipole moment [59], good formability [60], and flexibility [61], thus, it has been considered as a good candidate for an effective dielectric in TENG. Until now, various key approaches have been applied to develop promising materials for the enhancement of the output performance, such as the work function, the dielectric constant, the functional group (−F), the frictional coefficient, the surface resistivity, the carrier density, the intrinsic carrier density, etc. Among the approaches, a high dielectric constant and high work function were mainly studied theoretically and mathematically. In virtue of the dielectric constant, the total transferred charge density (*σ*′) in the metal-polymer system can be expressed by [62]

(1)  σ′=−σ0dgapdgap+ddielectricεdielectric 

where *σ*_0_ is the triboelectric surface charge density at the equilibrium state, *d_gap_* and *d_dielectric_* are the gap distance and the thickness of the dielectric films, and *ε_dielectric_* is a dielectric constant of the dielectric films. Thus, the charge density will be increased by the increase of the dielectric constant. The charge density is also influenced by the surface chemical potential difference between the two materials, according to the following general equation [27]

(2)  σ=[(W−E0)e](1+tεz)tεε0+(1/Ns(E)¯e2)(1+tεz) 

where *W* − *E*_0_ is the difference in the effective work functions between the two materials, *e*, *t*, *ε*, *ε*_0_, *z*, and *N_s_*(*E*) are the charge of an electron, distance of space, relative permittivity of the dielectric, vacuum permittivity of free space, thickness of the dielectric film, and the averaged surface density of states. This indicates that as the work function increases, the surface charge density accordingly increases. However, despite the above-detailed studies, the charge density by the artificial charge injection is practically limited and easily lost at ambient conditions. It implies that dielectric materials that can efficiently accept many charges by physical contact need to be developed.

## 2. Polyvinylidene Fluoride (PVDF) as an Effective Dielectric in Triboelectric Nanogenerators

PVDF, a highly non-reactive polymer with a strong piezoelectric response and low acoustic impedance, has been regarded as one of the most popular polymers in energy harvesting technologies. It also shows a good flexible and mechanical properties, making it a good candidate as an effective dielectric in triboelectric nanogenerator. It is a linear homopolymer produced by the repetition of CH_2_-CF_2_ monomers, crystallized into four possible conformations named as the α, β, γ, and δ phases. In general, they possess a permanent dipole moment. When the dipoles are aligned in one direction via mechanical stretching or an electrical poling process under a high electric field, the highest dipole moment can be obtained and this type of structure is known as β-phase PVDF film. The β-phase PVDF structure of PVDF has fluorine atoms and hydrogen atoms in the opposite direction, forming a high dipole density. Thus, it intrinsically has a high dielectric constant (~10), compared to other polymers. In order to additionally the dielectric constant, various ferroelectric particles such as BaTiO_3_ [63], ZnSnO_3_ [47], and SrTiO_3_ [64] are blended and functional groups such as -TrFE [28], -HFP [29], and -PtBA [48] are grafted via copolymerization technique. These fluorine functional groups exhibit large electron affinity, increasing the charges-accepting characteristics from the metal during the instantaneous contact. With this material synthesis by exploiting intrinsic properties of ferroelectric materials, new dielectric materials should be developed to increase the energy conversion efficiency for sustainable self-powered devices.

### 2.1. PVDF-Based Polymers as an Effective Dielectric

**Figure 3:**
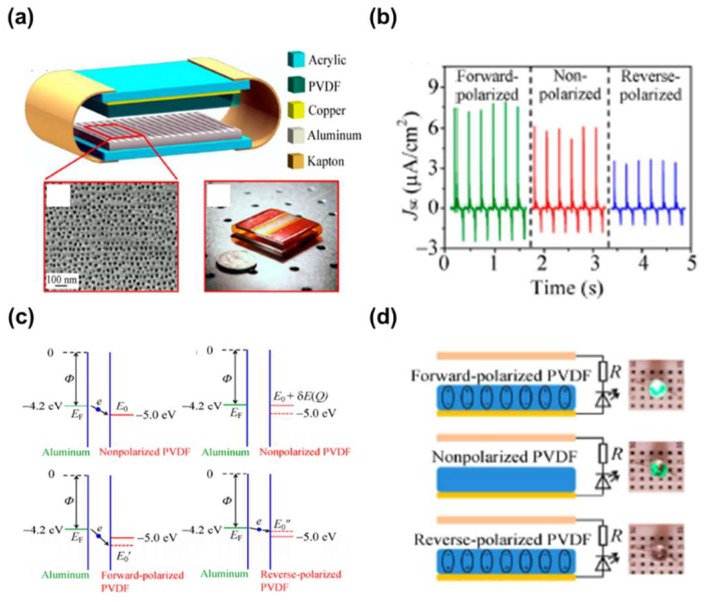
(**a**) The schematic of the TENG with a double-layer structure. A SEM image of nanopores on an aluminum foil. Photograph of a TENG. (**b**) *J_sc_* of TENGs fabricated using different types of polyvinylidene fluoride (PVDF) films under a periodic compressive force of around 50 N applied by an electric shaker at a frequency of 4 Hz. (**c**) Schematic energy band diagram illustrating the process when electrons tunnel between E_F_ and E_0_. The electric field developed by charge transfer raises the energy of the electrons in the PVDF’s surface states by δ*E*(*Q*). The positive bond charges of the forward-polarized PVDF reduce the characteristic energy level of PVDF to E_0′_. The negative bond charges of the reverse-polarized PVDF increase the characteristic energy level of PVDF to E_0_″. (**d**) Circuit diagrams and the different performances among TENGs fabricated using different types of PVDF when they were used as direct power source for LED bulbs. Reproduced with permission from Wang et al., *Nano Research* **2015**, *15*, 990–997. Copyright 2015 Springer Nature.

The Wang group, for the first time, used PVDF as a dielectric without any modifications [46]. As shown in Figure 3a, the TENG consists of two layers, a nanoporous aluminum (Al) foil and a PVDF thin film deposited on a copper substrate with Kapton films of a thickness of 125 μm as a spacer. During the cycled contact under a compressive force of around 50 N, the TENG produced a short-circuit current density (*J_sc_*) of 6.13 μA/cm^2^ (Figure 3b). After forward and reverse polarization, the *J_sc_* increased to 8.34 μA/cm^2^ and 4.83 μA/cm^2^, respectively. By averaging the instantaneous peaks, the forward-polarized TENG showed an output performance 3 times larger than that of the reverse-polarized TENG. They explained the enhancement in terms of the surface potential of the PVDF thin film modified by the polarization process, as shown in Figure 3c. When the film was forward-polarized, the net dipole moment was increased because of the arrangement of the dipoles along the same direction. This reduced the characteristic energy level of the PVDF, thereby increasing the potential difference with the Fermi level of Al. This resulted in an enhancement of the charge-transfer characteristics between the PVDF and the Al electrode. The light-emitting diode (LED) connected to the forward-polarized TENG was much brighter than that of the reverse-polarized TENG (Figure 3d). This shows the experimental evidence of the relationship between the work function difference between the PVDF and Al, and the electric outputs of the TENG.

**Figure 4:**
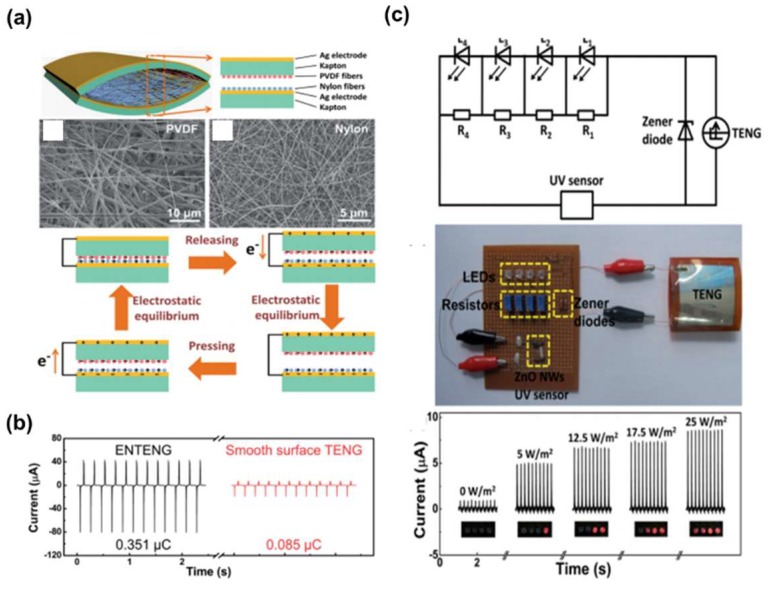
(**a**) The schematic of the TENG based on electrospun nanowires (ENTENG). SEM images of the electrospun PVDF and nylon nanofibers. The charge distribution in the device and the flowing direction in the circuit when the device is pressed and released. (**b**) The output current of the ENTENG and the output current of the smooth surface TENG with a similar device structure. (**c**) The schematic and optical images of the ultraviolet radiation (UVR) level detection system powered by an ENTENG. Current flowing across the UV sensor and the optical images of the LEDs at UV intensities of 0, 5, 12.5, 17.5, and 25 Wm^−2^. Reproduced with permission from Jing et al., *Nanoscale* **2014**, *6*, 7842–7846. Copyright 2014 The Royal Society of Chemistry.

The PVDF nanofibers of approximately 790 nm in diameter by the electrospinning method were also prepared, deposited on Kapton film, and contacted with the nylon nanofibers as shown in Figure 4a [65]. Based on the triboelectric series, negative charges are transferred to the PVDF from the nylon, creating instantaneous electrons flow through the external circuit between two electrodes. The TENG produced an instantaneous output power of 26.6 W/m^2^ and it was demonstrated that the nanofibers were so effective in increasing the output current of TENGs, compared with the films as shown in Figure 4b. Thus, electrospinning, as a fiber production method, is appropriate for fabricating roughened surfaces to enhance the output power. The nanofibers also seem so flexible, quite helpful for harvesting wearable energy sources. The TENG was also connected to Zener diodes and a ZnO nanowire sensor to demonstrate the possibility of the TENG as a power source for a self-powered UV sensor, which was successfully proven (Figure 4c).

**Figure 5:**
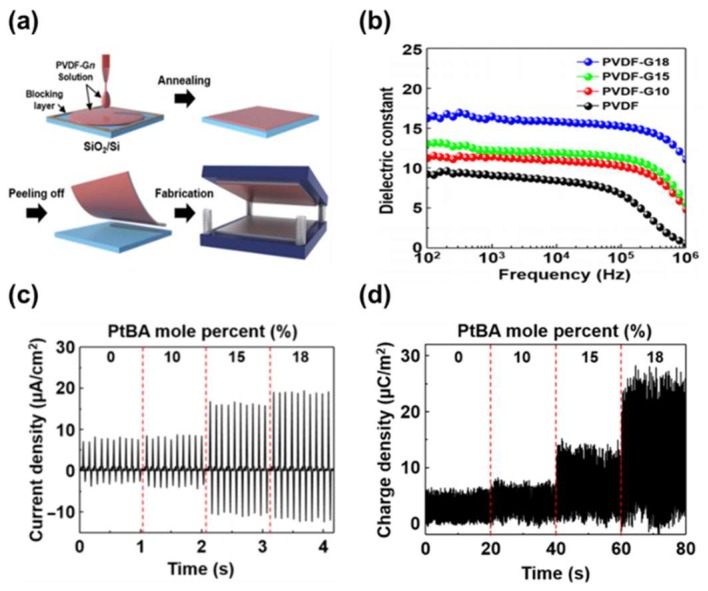
(**a**) The schematic diagrams of the fabrication process for the PVDF-Gn–based TENGs. (**b**) Frequency dependence of the dielectric constant values with various PtBA mole percentages ranging from 0 to 18%. (**c**) Output current densities generated by the PVDF-based TENGs as a function of the PtBA mole percentage ranging from 0 to 18%. (**d**) Charge densities generated by the PVDF-based TENGs as a function of the PtBA mole percentage ranging from 0 to 18%. Reproduced with permission from Baik et al., *Sci. Adv.* **2017**, *3*, 1500661. Copyright 2017 American Association for the Advancement of Science.

Very recently, there has been some progress in developing PVDF-based polymers as an effective dielectric in TENG. Baik group successfully synthesized poly (tert-butyl acrylate) (PtBA)-grafted PVDF copolymers through an atom transfer radical polymerization technique and demonstrated that the copolymers were very effective in increasing output performance, compared with pristine PVDF [48]. Unlike the PVDF copolymers such as P(VDF-TrFE), P(VDF-HFP) was mainly composed of β-phase. The copolymer was reported to be mostly composed of *α* phases with enhanced dipole moments by π-bonding and polar characteristics of the ester functional groups in the PtBA, bringing about the increase of the dielectric constant by approximately twice. In order to understand the effect of the dielectric constant on the output signal of the TENG, a very flat PVDF surface was made by the peeling-off process, as shown in Figure 5a. As the grafting ratio increased to 18%, the dielectric constant was increased from 8.6 to 16.5 in the frequency range of 10^2^–10^5^ Hz. This was attributed to the increase of the net dipole moment, as shown in Figure 5b. This increase in the dielectric constant enhanced the charge density that can be accumulated on the surface, generating the output voltage and current density of 64.4 V and 18.9 μA/cm^2^, respectively, twice enhancement in both, compared to pristine PVDF based nanogenerator as shown in Figure 5c. To prove the results, they measured the accumulated surface charges density with different dielectric constant values, which showed an excellent agreement with the measured output current values as shown in Figure 5d. This paper, for the first time, experimentally proved that the output power linearly increased with the dielectric constant. However, the output power was still low because of the low dielectric constant value.

### 2.2. PVDF-Based Polymers Hybridized with Inorganic Materials

**Figure 6:**
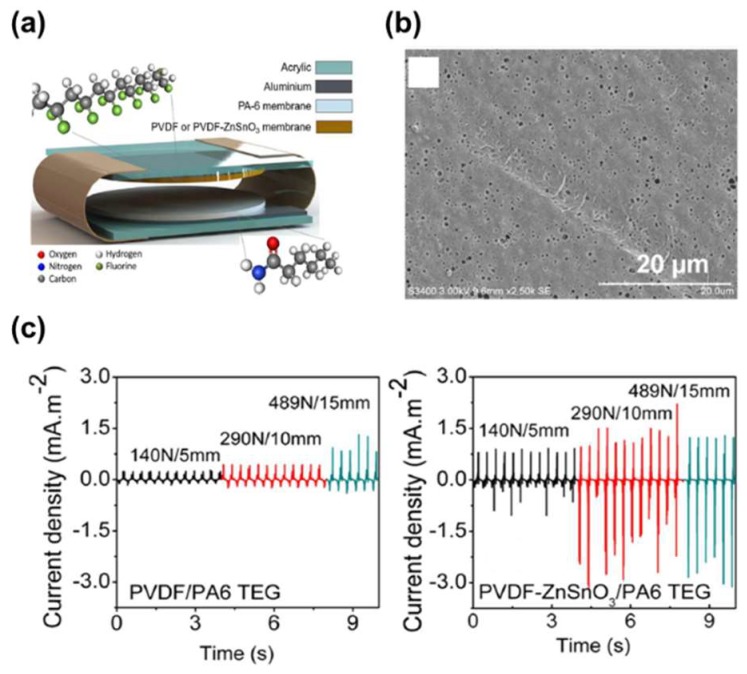
(**a**) The structure of the triboelectric nanogenerator (TEG) showing the position of the various components. (**b**) Scanning electron microscopy images of the surface and thickness cross-section of the PVDF-ZnSnO_3_ membrane. (**c**) Short circuit current values for PVDF/PA6 TEG and PVDF-ZnSnO_3_/PA6 TEG. Reproduced with permission from Luo et al., *Nano Energy* **2016**, *30*, 470–480. Copyright 2016 Elsevier.

As mentioned above, despite the many advantages of PVDF polymers as a dielectric, it still had a low dielectric constant, thereby it limits the output power of the TENG. Thus, PVDF polymers embedded with inorganic nanoparticles have been utilized as dielectrics in TENGs. Zinc stannate (ZnSnO_3_) materials with a strong piezoelectric response and huge spontaneous polarization value have been widely used as a candidate in polymers—such as PDMS—in which most of those studies have been focused on piezoelectricity or the piezoelectric nanogenerator. Recently, a TENG with the vertical contact-separation mode which consisted of ZnSnO_3_ nanocubes-PVDF composites and a polyamide-6 (PA6) membrane was reported, as shown in Figure 6a. The high dispersion of ZnSnO_3_ in the composites was obtained by increasing the shear speeds during the compounding process. By the phase inversion process, the composites showed that a porous structure only exists on the surface in which the pore size is in the range of 0.5–1.0 μm, as shown in Figure 6b. Compared with the pristine PVDF, the composites also showed the increase of the dielectric permittivity corresponding to a higher polarization and a higher piezoelectric coefficient d_33_ of −65 pm/V. In the TENG, the PA6 membrane was attached by using an adhesive aluminum tape electrode on one side and a PVDF or PVDF-ZnSnO_3_ composite attached to the opposite side using aluminum tape. The composites are contacted with a PA6 membrane, showing an increase of the instantaneous output signals such as 520 V and 2.7 mA/m^2^ in output voltage and output current, respectively. It corresponds to an enhancement of 70% and 200%, respectively as shown in Figure 6c.

**Figure 7:**
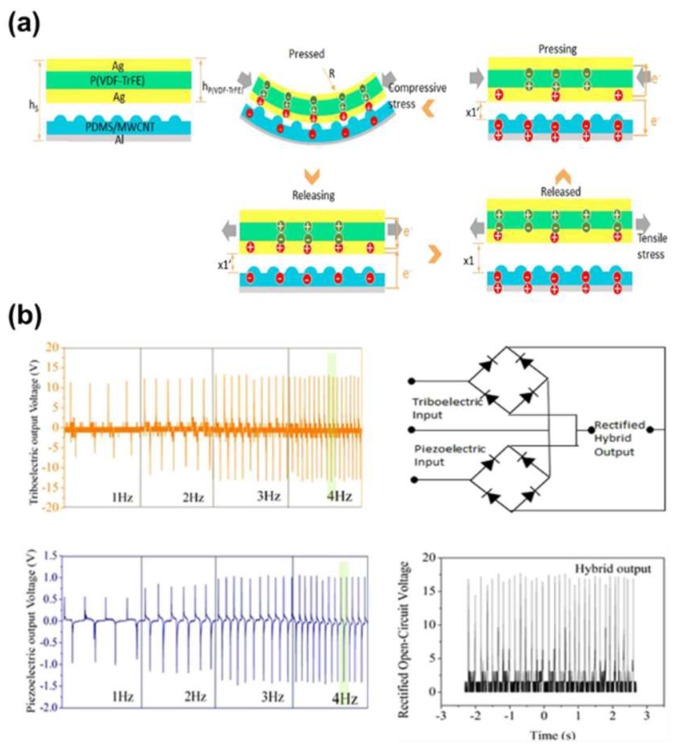
(**a**) The cross-section structural schematic; Charge distribution of triboelectric and piezoelectric effect during force pressing and releasing. Inside of the picture, 0 < x_1_′ < x_1_, h_s_ is the summation height of TPENG, h_P (VDF-TrFE)_ is the height of P(VDF-TrFE) film, R is the stretching radius. (**b**) The open-circuit performance of the triboelectric and piezoelectric under the force of 5 N as a function of frequency. The circuit diagram of the hybrid output in which the piezoelectric and triboelectric outputs are combined in parallel. Hybrid open-circuit output voltage. Reproduced with permission from Luo et al., *Sci. Rep.* **2016**, *6*, 36409. Copyright 2016 Springer Nature.

In addition to the inorganic nanoparticles, PVDF based nanocomposites composed of PVDF-PBA-MWCNTs (PCNTs) were successfully synthesized, as shown in Figure 7a [66]. With the incorporation of PCNTs into the PVDF matrix, the crystalline phase transition significantly occurred from the *α*-phase to the *β*-phase, meaning that the *β*-phase was enhanced. It was explained in terms of the enhancement of compatibility of the PCNTs with CF_2_ groups in the PVDF by the carboxylic acid groups, increasing the nucleation sites for the *β*-phase. Under the mechanical deformation at 3 Hz, the PVDF-10PCNT composite showed an output performance enhancement of almost 8 times compared to the pristine PVDF film by the physical contact with the Al film, as shown in Figure 7b.

## 3. PVDF Based Applications

In addition to power generation, PVDFs have been also extensively studied in sensor applications by many researchers. Actually, PVDFs have attracted much attention as an important material in various applications for its exceptional properties such as its high thermal stability, outstanding chemical resistance, low acoustic impedances, low permitivities, flexibility, and membrane forming properties. Additionally, PVDF is pyroelectric, meaning that it has a higher temperature dependent performance compared to other ceramic based sensors. With these excellent properties of PVDF, human skin-inspired multimodal e-skins were developed by mimicking various structures and functions of the elaborate sensory system in human finger-tips [67]. Here, polymer composites consisted of PVDF and reduced graphene oxide (rGO) were synthesized and a micro-structured ferroelectric skin was fabricated by mimicking the sensor system in human fingertips, in which the piezoresistive change between interlocked microdome arrays of rGO/PVDF composites was measured. This e-skin was also multifunctional and could detect pressure, vibration, and temperature with quite high sensitivities. A free-standing ZnO nanorods/PVDF composites are synthesized to fabricate a multifunctional tactile sensor that can independently measure the pressure and temperature [68]. Due to the enhanced β-phase of the PVDF film by the ZnO nanostructures, the minimum detectable pressure of the sensor was about 10 Pa and could detect temperatures in the range of 20–120 °C.

However, most of the research on the sensor based PVDF relies on the piezoelectric/ferroelectric properties of the material. Recently, there have been some reports on the sensing performance based on triboelectric effects in which the PVDF-based materials were used as positively charged layers to utilize its piezoelectric properties, thus, to fabricate the hybrid nanogenerator [69]. To enhance the flexibility and stretchability, and thus, the contact uniformity, the PVDF nanofibers were fabricated by the electrospinning technique. The hybridization was very effective in increasing the output voltage when both outputs were combined in parallel. It was also reported that it could exhibit a high sensitivity of 0.068 V/kPa in the range of 100 and 700 kPa.

## 4. Conclusions

Herein, various TENG technologies including the device structures, operation modes, a few functional materials, as well as the fundamental issues such as the working mechanism, and the contribution to the substantial increase of the output power up to several tens of mW/cm^2^, were reported. We also reviewed PVDF-based materials as a promising candidate for efficient dielectrics in TENGs with a high output performance. PVDFs have many advantages such as their excellent mechanical properties (e.g., flexibility) and molecular structures showing high ferroelectric properties and high electron affinities due to the C-F bonds, making it possible to synthesize new dielectrics for further increases in the output power of TENGs. Up to now, although there were a few, not many reports on the synthesis of the PVDF-based materials exist as most of the papers have focused on the increase of the dielectric constant via the copolymerization techniques or the hybridization with inorganic nanomaterials. This strategy, together with the increase of surface roughness, was proven to be very effective in increasing the output power of the TENG. However, despite many efforts, the output power may not be enough for the realization of self-powered systems. From the material’s aspect, the efficiency of the charge transfer to the dielectric and the capability of holding the charge in the dielectric need to be improved. The fabrication of TENGs based on the multilayered dielectric film has been reported as being quite effective in increasing the output power. Thus, it is thought that the TENGs will be used as portable power sources for low-power electronic devices such as sensors because of the low outputs. The TENG can also be utilized to fabricate physical sensors which are capable of sensing strain/stress, motion, acceleration etc. By virtue of increased output power and advanced technologies, self-powered systems consisting of some devices with high-power consumption will also be realized in the near future. This review can be useful and helpful for developing effective dielectrics of energy harvesters and sensor which are able to supply sufficient energy with portable electronic devices and to be highly sensitive and multifunctional.

## Figures and Tables

**Table 1:** The areal power densities, output currents, and charge densities of various triboelectric nanogenerators (TENGs) reported for the last 5 years.

	Positively Charged Materials	Negatively Charged Materials	Working Mode	Output Power (mW/cm^2^)	Charge Density (μC/m^2^)
#1 [1]	PET	Kapton	Vertical-contact	0.00036	-
#2 [2]	PET	PDMS	Vertical-contact	0.00234	-
#3 [21]	Al	PDMS	Vertical-contact	3.56	-
#4 [22]	Au	PDMS	Vertical-contact	31.3	594.2 (calculated)
#5 [23]	Nylon	PTFE	Lateral-sliding	0.53	59
#6 [24]	Al	FEP	Vertical-contact	31.5	240
#7 [46]	Al	PVDF	Vertical-contact	0.26	360.2
#8 [12]	Cu	PTFE	Freestanding	50	323
#9 [47]	Al	ZnSnO3-PVDF (composites)	Vertical-contact	3	101.3
#10 [25]	Cu	Kapton	Lateral-sliding	13.2	-
#11 [26]	Al	PDMS	Vertical-contact	46.8	270
#12 [48]	Al	PVDF-Gn	Vertical-contact	2.6	23
